# Prognostic and clinicopathological significance of systemic inflammation response index in patients with hepatocellular carcinoma: a systematic review and meta-analysis

**DOI:** 10.3389/fimmu.2024.1291840

**Published:** 2024-02-26

**Authors:** Sunhuan Zhang, Zhining Tang

**Affiliations:** Clinical Laboratory, Huzhou Central Hospital, Affiliated Central Hospital of Huzhou University, The Fifth School of Clinical Medicine of Zhejiang Chinese Medical University, Huzhou, Zhejiang, China

**Keywords:** SIRI, meta-analysis, hepatocellular carcinoma, prognosis, evidence-based medicine

## Abstract

**Background:**

It is unclear whether the systemic inflammation response index (SIRI) can predict the prognosis of patients with hepatocellular carcinoma (HCC). Consequently, the present study focused on systematically identifying the relationship between SIRI and the prognosis of patients with HCC through a meta-analysis.

**Methods:**

Systematic and comprehensive studies were retrieved from PubMed, Web of Science, Embase, and the Cochrane Library from their inception to August 10, 2023. The role of SIRI in predicting overall survival (OS) and progression-free survival (PFS) in HCC was determined using pooled hazard ratios (HRs) and 95% confidence intervals (CIs). Odds ratios (ORs) and 95% CIs were pooled to analyze the correlations between SIRI and the clinicopathological features of HCC.

**Results:**

Ten articles involving 2,439 patients were included. An elevated SIRI was significantly associated with dismal OS (HR=1.75, 95% CI=1.52–2.01, p<0.001) and inferior PFS (HR=1.66, 95% CI=1.34–2.05, p<0.001) in patients with HCC. Additionally, according to the combined results, the increased SIRI was significantly related to multiple tumor numbers (OR=1.42, 95% CI=1.09–1.85, p=0.009) and maximum tumor diameter >5 cm (OR=3.06, 95% CI=1.76–5.30, p<0.001). However, the SIRI did not show any significant relationship with sex, alpha-fetoprotein content, Child-Pugh class, or hepatitis B virus infection.

**Conclusion:**

According to our results, elevated SIRI significantly predicted OS and PFS in patients with HCC. Moreover, the SIRI was significantly associated with tumor aggressiveness.

**Systematic review registration:**

https://inplasy.com/inplasy-2023-9-0003/, identifier INPLASY202390003.

## Introduction

Primary liver cancer ranks sixth among cancers in terms of morbidity and is the third most common cause of cancer-associated mortality worldwide (1). As estimated by GLOBCAN, 905,677 new liver cancer cases and 830,180 liver cancer-associated deaths were reported globally in 2020 (1). Hepatocellular carcinoma (HCC), the most frequent subtype of liver cancer, affects approximately 75% of patients worldwide (2). Approximately 72% of the HCC cases are reported in Asia (over 50% in China), and 10%, 7.8%, 5.1%, 4.6%, and 0.5% in Europe, Africa, North America, Latin America, and Oceania, respectively (3). In general, surgery, local thermal ablation, liver transplantation (LT), transcatheter arterial chemoembolization (TACE), and systemic therapy are the main treatments for HCC and have shown efficacy in reducing the mortality rates of HCC (4). Despite this, the long-term survival rates of patients remain unsatisfactory, and recurrence rates are high (5). Among patients with localized or metastatic HCC, the 5-year overall survival (OS) rate is < 10% (6). In addition, up to 70% of patients experience recurrence after undergoing treatment with a curative intent (6). Therefore, the identification of effective prognostic biomarkers is pivotal for risk stratification and adjunctive treatment development in patients with HCC.

Accumulating evidence suggests that immune responses and inflammation influence tumor progression and metastasis (7). Many inflammatory blood-based indices, such as the neutrophil-to-lymphocyte ratio (NLR) (8), platelet-to-lymphocyte ratio (PLR), albumin-to-globulin ratio (AGR) (9), lymphocyte-to-monocyte ratio (LMR) (10), and C-reactive protein-to-albumin ratio (CAR) (11), are significant prognostic markers of different cancer types. The systemic inflammation response index (SIRI) is a novel hematologic parameter that was first proposed in 2016 (12) and is determined using the following formula: SIRI = (neutrophil × monocyte)/lymphocyte count. SIRI has been widely suggested to exhibit a significant and powerful value in predicting solid tumors such as bladder cancer (13), non-small cell lung cancer (NSCLC) (14), gastric cancer (15), breast cancer (16), and ovarian cancer (17). The impact of the SIRI on predicting HCC prognosis has also been explored; however, no consistent findings have been found (18–27). In certain studies, elevated SIRI was found to be a significant prognostic marker of HCC (23–25), while other studies have shown no obvious relationship between SIRI and HCC survival (21, 27). This meta-analysis aimed to accurately identify the prognostic effects of SIRI in patients with HCC. Additionally, the relationship between SIRI and clinicopathological features of HCC was explored.

## Materials and methods

### Study guideline

The present meta-analysis was conducted in accordance with the guidelines of the Preferred Reporting Items for Systematic Reviews and Meta-Analyses (PRISMA) (28). Our meta-analysis protocol was registered in INPLASY (registration number: INPLASY202390003) and can be found at https://inplasy.com/inplasy-2023-9-0003/.

### Ethics statement

Data in this study were extracted from publications, and, as a result, ethical approval or patient consent was waived.

### Literature search

The PubMed, Web of Science, Embase, and Cochrane Library databases were comprehensively searched from their inception to August 10, 2023, using the following search terms: (systemic inflammation response index or systemic inflammatory response index) and (hepatocellular carcinoma, hepatocellular cancer, HCC, or liver cancer). A detailed search strategy for each database is provided in **Supplementary Data Sheet 1**. The language used in this study was English. To identify additional eligible articles, we manually searched the reference lists of each retrieved article.

### Inclusion and exclusion criteria

Articles satisfying the following criteria were recruited: (1) HCC was diagnosed based on pathology or histology; (2) studies investigating the relationship between SIRI and prognosis of patients with HCC; (3) those with available or calculable hazard ratios (HRs) and 95% confidence intervals (CIs); (4) those mentioning threshold SIRI; (5) those reporting survival outcomes such as OS, disease-free survival (DFS), progression-free survival (PFS), or cancer-specific survival (CSS); and (6) English language articles. The following studies were excluded: (1) meeting abstracts, reviews, comments, letters, and case reports; (2) animal studies; and (3) those including overlapping patients.

### Data extraction and quality assessment

The studies were reviewed and data were independently extracted from qualified studies by two reviewers (SZ and ZT). Any dispensaries were resolved through negotiation until a consensus was reached. The following information was collected: first author, publication year, country, study design, sample size, age, sex, study center, study period, Child-Pugh class, treatment, threshold, threshold selection method, survival outcomes, survival analysis type, follow-up, HRs, and 95%CIs for survival outcomes. If eligible studies underwent propensity score matching (PSM) analysis, the data for the entire population were extracted and analyzed to avoid selection bias. OS and PFS were defined as the primary and secondary survival outcomes, respectively. Two researchers (SZ and ZT) evaluated the literature quality using the Newcastle-Ottawa Scale (NOS) (29) and crosschecked our results. The NOS assesses literature quality from three perspectives: selection, comparability, and outcome measurement. The NOS score ranges from 0 to 9, with studies scoring ≥ 6 points considered to be of high quality.

### Statistical analysis

The significance of the SIRI in predicting the OS and PFS of patients with HCC was evaluated based on the combined HRs and 95% CI. Interstudy heterogeneity was evaluated using the Higgins I^2^ statistic and Cochran’s Q test. The random-effects model was applied when I^2^ was >50% or P was <0.1; otherwise, the fixed-effects model was used. Diverse factor-stratified subgroup analyses were conducted to identify sources of heterogeneity. The relationships between SIRI and the clinicopathological characteristics of HCC were analyzed using combined odds ratios (ORs) and 95% CIs. A sensitivity analysis was used to evaluate the consistency of the findings. Meta-regression was conducted to evaluate the effect of clinicopathological factors on the overall results. Begg’s test, funnel plots, and Egger’s test were used to examine possible publication bias. Stata software (version 12.0; Stata Corp., College Station, TX, USA) was used for graph generation and statistical analyses. Statistical significance was set at P<0.05.

## Results

### Study selection process

**Figure 1:**
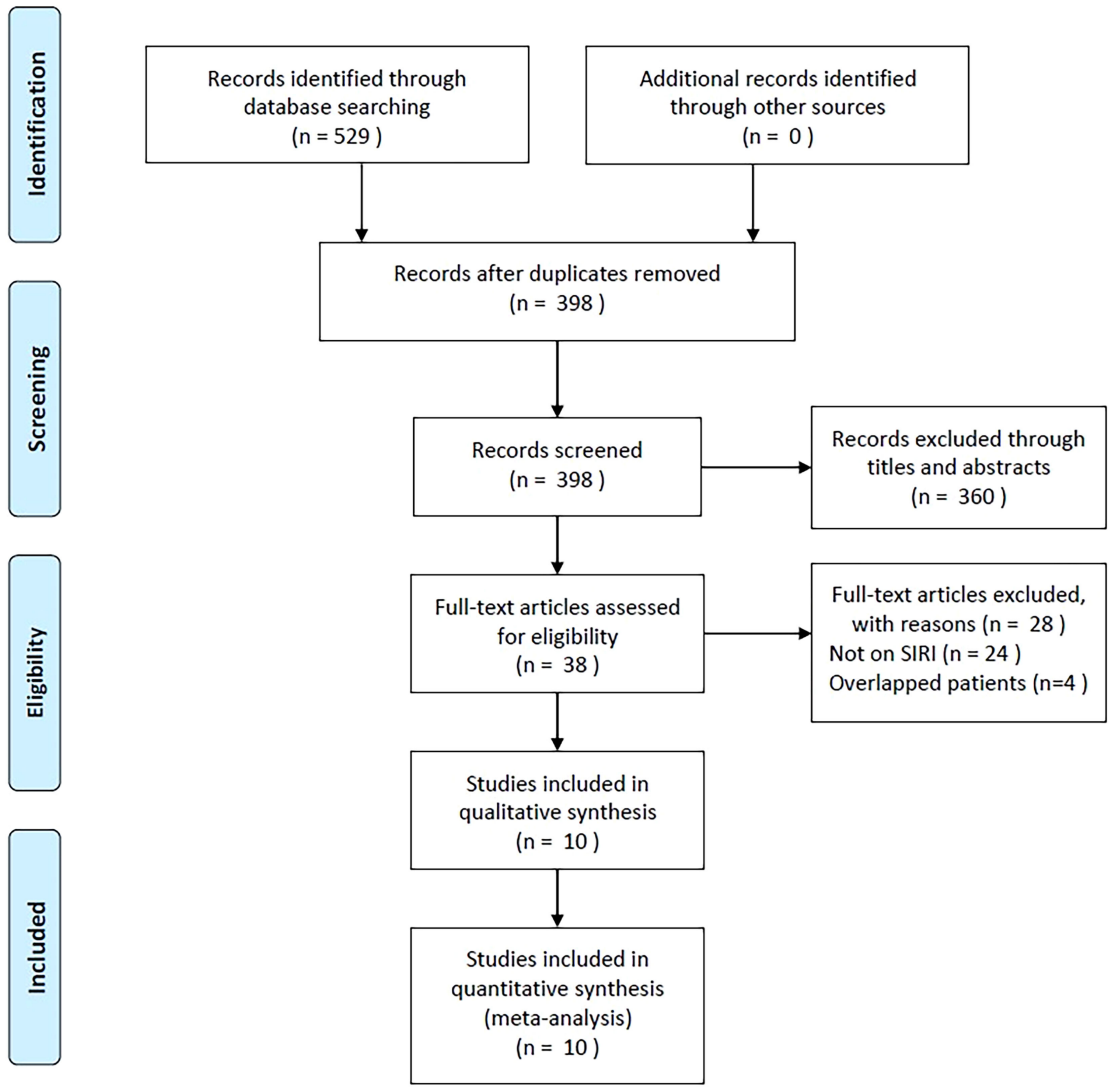
PRISMA flowchart of the established screening strategy.

As shown in **Figure 1**, 529 studies were identified through primary literature retrieval, and 398 records were retained following the removal of duplicates. Subsequently, 360 articles were discarded by title and abstract screening owing to their irrelevance. Then, the full texts of 38 articles were examined, and another 28 were excluded because they did not focus on SIRI (n=24) or recruited overlapping patients (n=4). Finally, the present meta-analysis recruited 10 articles involving 2,439 patients (18–27) (**Figure 1**; **Table 1**).

**Table 1:** Baseline characteristics of included studies in this meta-analysis.

Study	Year	Country	Sample size	Study design	Study period	Gender (M/F)	Age (year) Median(range)	Treatment	Child-Pugh class	Study center	Cut-off value	Cut-off selection	ROC methods	Survival outcomes	Survival analysis	Follow-up (month) Median(range)	NOS score
Xu, L.	2017	China	351	Retrospective	2006-2013	300/51	53.9	Surgery	A-B	Single center	1.05	ROC curve	NR	OS	Multivariate	1-100	8
Cinkir, H. Y.	2020	Twurkey	80	Retrospective	2011-2018	67/13	69(29-83)	Sorafenib	A-B	Single center	2.2	ROC curve	Youden index	OS	Univariate	7.8(1.2-38.7)	7
Wang, T. C.	2021	China	194	Retrospective	2014-2019	174/20	56.5	TACE	A-B	Multicenter	0.88	ROC curve	NR	OS, PFS	Univariate	1-80	9
Wu, Y.	2021	China	161	Retrospective	2011-2018	141/20	56.2	Surgery	A-B	Single center	1.03	ROC curve	Youden index	PFS	Multivariate	1-120	8
Zou, Y.	2021	China	370	Retrospective	2013-2019	325/45	54.2	Surgery	A-B	Single center	1.17	ROC curve	NR	PFS	Multivariate	1-96	8
Xin, Y.	2022	China	403	Retrospective	2012-2017	328/75	58(30-80)	RFA	A-B	Single center	1.36	X-tile	NR	OS, PFS	Univariate	44.5(12.6-95.0)	8
Zhao, M.	2022	China	352	Retrospective	2013-2020	290/62	58(26-86)	Sorafenib/ICIs	A-C	Single center	1.64	ROC curve	NR	OS, PFS	Univariate	1-100	7
Cui, S.	2023	China	218	Retrospective	2010-2020	197/21	53.9	LT	A-C	Single center	1.25	ROC curve	NR	OS, PFS	Multivariate	39.4	8
Mao, S.	2023	China	148	Retrospective	2016-2020	125/23	58.6	TACE	A-B	Single center	1.04	ROC curve	NR	PFS	Multivariate	13(1-36)	7
Wenpei, G.	2023	China	162	Retrospective	2013-2016	125/37	≤60 y: 107 >60 y: 55	Surgery	A-B	Single center	0.785	ROC curve	Youden index	PFS	Multivariate	1-24	8

M, male; F, female; TACE, transcatheter arterial chemoembolization; RFA, radiofrequency ablation; LT, liver transplantation; ICIs, immune checkpoint inhibitors; ROC, receiver operating characteristic; OS, overall survival; PFS, progression-free survival; NOS, Newcastle-Ottawa Scale.

### Included study features

**Table 1** presents the baseline characteristics of the selected articles. The publication years of these articles ranged between 2017 and 2023 and all had a retrospective design. Nine studies were conducted in China (18, 20–27) while one was conducted in Turkey (19). The sample size across the selected articles ranged from 80 to 403 (median, 206). We therefore selected 200 for subgroup analysis of sample size. Four studies treated patients with HCC with surgery (18, 21, 22, 27), two studies used TACE treatment (20, 26), and one each used sorafenib (19), radiofrequency ablation (RFA) (23), sorafenib/immune checkpoint inhibitors (ICIs) (24), and LT (25). Nine studies were single-center studies (18, 19, 21–27) and one was a multicenter study (20). The threshold SIRI was 0.785–2.2 (median, 1.11). We, therefore, used 1.1 for subgroup analysis of cut-off value in the following analyses. Nine articles used the receiver operating characteristic (ROC) curve to determine the best threshold (18–22, 24–27) and one study applied the X-tile software (23). Six studies reported the significance of SIRI in the OS prediction of HCC (18–20, 23–25) and eight studies reported the association between SIRI and PFS (20–27). Six studies reported HRs and 95%CIs using multivariate analysis (18, 21, 22, 25–27) and four studies used univariate analysis (19, 20, 23, 24). The NOS scores of all eligible studies were 7–9, suggesting a high quality (**Table 1**).

### SIRI and OS

**Figure 2:**
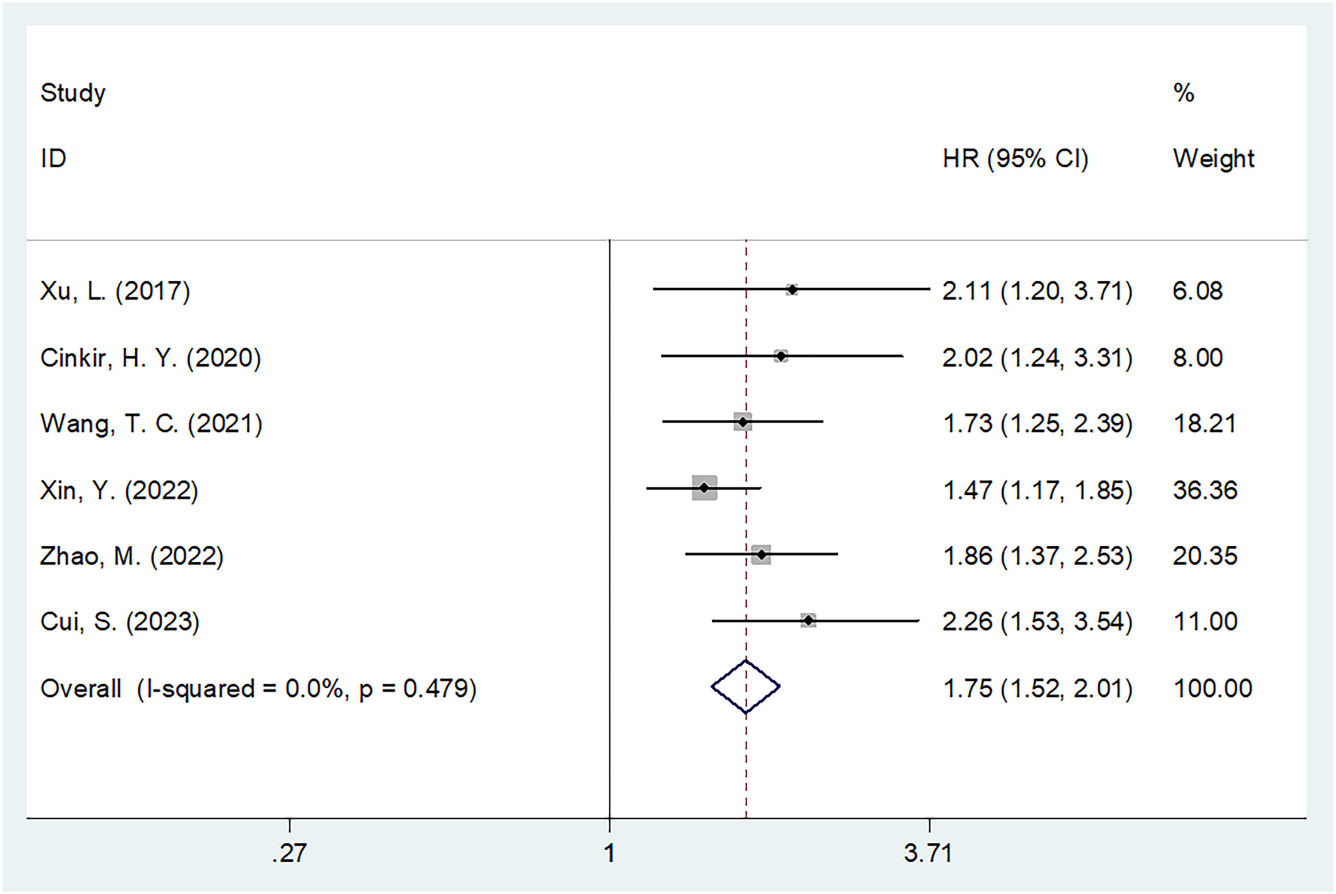
Forest plots of the prognostic role of SIRI for OS in patients with HCC.

Six studies involving 1,598 patients (18–20, 23–25) provided data on the significance of SIRI in predicting OS in HCC. There was no obvious heterogeneity (I^2^ = 0, P=0.479); therefore, we adopted a fixed-effects model. As shown in **Table 2** and **Figure 2**, the HR was 1.75 (95% CI=1.52–2.01, p<0.001), suggesting a relationship between elevated SIRI and dismal OS in patients with HCC. Subgroup analysis revealed that the predictive role of SIRI in OS remained consistent across various factors including country, treatment, sample size, study center, threshold, threshold selection method, or survival analysis type (**Table 2**).

**Table 2:** Subgroups of the prognostic value of SIRI for OS in patients with HCC.

Subgroups	No. of studies	No. of patients	Effects model	HR (95%CI)	p	Heterogeneity I^2^(%) Ph		Meta-regression p
Total	6	1,598	Fixed	1.75(1.52-2.01)	<0.001	0	0.479	
Country								0.305
Turkey	1	80	–	2.02(1.24-3.30)	0.005	–	–	
China	5	1,518	Fixed	1.72(1.49-1.99)	<0.001	3.4	0.387	
Sample size								0.649
<200	2	274	Fixed	1.81(1.38-2.38)	<0.001	0	0.602	
≥200	4	1,324	Fixed	1.72(1.47-2.03)	<0.001	27.5	0.247	
Treatment								0.827
Surgery	1	351	–	2.11(1.20-3.71)	0.009	–	–	
TACE	1	194	–	1.73(1.25-2.39)	0.001	–	–	
Sorafenib or Sorafenib/ICIs	2	432	Fixed	1.91(1.47-2.47)	<0.001	0	0.783	
LT	1	218	–	2.26(1.49-3.43)	<0.001	–	–	
RFA	1	403	–	1.47(1.17-1.85)	0.001	–	–	
Study center								0.528
Single center	5	1,404	Fixed	1.75(1.50-2.04)	<0.001	11.1	0.343	
Multicenter	1	194	–	1.73(1.25-2.39)	0.001	–	–	
Cut-off value								0.283
<1.1	2	545	Fixed	1.82(1.37-2.41)	<0.001	0	0.545	
≥1.1	4	1,053	Fixed	1.73(1.47-2.02)	<0.001	25.8	0.257	
Cut-off selection								0.714
ROC curve	5	1,195	Fixed	1.93(1.62-2.29)	<0.001	0	0.883	
X-tile	1	403	–	1.47(1.17-1.85)	0.001	–	–	
Survival analysis								0.927
Univariate	4	1,029	Fixed	1.67(1.43-1.94)	<0.001	0	0.522	
Multivariate	2	569	Fixed	2.20(1.58-3.09)	<0.001	0	0.851	

SIRI, systemic inflammation response index; OS, overall survival; HCC, hepatocellular carcinoma; TACE, transcatheter arterial chemoembolization; RFA, radiofrequency ablation; LT, liver transplantation; ICIs, immune checkpoint inhibitors; ROC, receiver operating characteristic.

### SIRI and PFS

**Figure 3:**
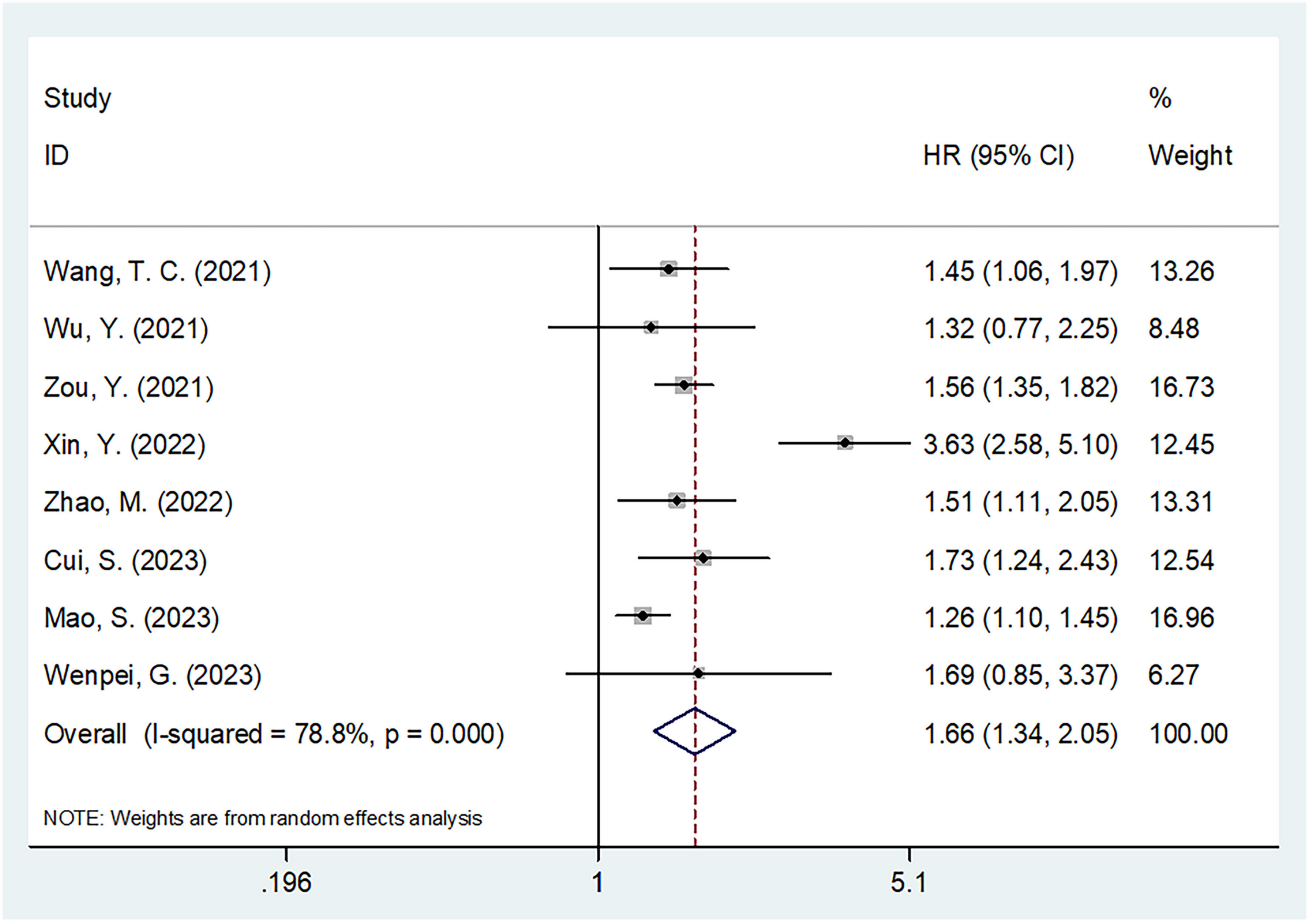
Forest plots of the prognostic role of SIRI for PFS in patients with HCC.

Altogether, eight articles comprising 2,008 patients (20–27) reported a correlation between SIRI and PFS in patients with HCC. Because of significant heterogeneity, we employed the random-effects model (I^2^ = 78.8%, P<0.001; **Table 3** and **Figure 3**). Based on the pooled results, an increased SIRI significantly predicted inferior PFS in HCC (HR=1.66, 95% CI=1.34–2.05, p<0.001; **Figure 3**; **Table 3**). Subgroup analysis indicated that elevated SIRI still significantly predicted PFS in HCC, which was unaffected by country, treatment, sample size, study center, threshold, threshold selection method, or survival analysis type (**Table 3**).

**Table 3:** Subgroups of the prognostic value of SIRI for PFS in patients with HCC.

Subgroups	No. of studies	No. of patients	Effects model	HR (95%CI)	p	Heterogeneity I^2^(%) Ph		Meta-regression p
Total	8	2,008	Random	1.66(1.34-2.05)	<0.001	78.8	<0.001	
Sample size								0.795
<200	4	665	Fixed	1.31(1.16-1.47)	<0.001	0	0.760	
≥200	4	1,343	Random	1.94(1.36-2.76)	<0.001	85.5	<0.001	
Treatment								0.891
Surgery	3	693	Fixed	1.55(1.35-1.79)	<0.001	0	0.810	
TACE	2	342	Fixed	1.29(1.14-1.47)	<0.001	0	0.433	
Sorafenib or Sorafenib/ICIs	1	352	–	1.51(1.11-2.05)	0.008	–	–	
LT	1	218	–	1.73(1.24-2.43)	0.001	–	–	
RFA	1	403	–	3.63(2.58-5.10)	<0.001	–	–	
Study center								0.460
Single center	7	1,814	Random	1.69(1.33-2.16)	<0.001	81.8	<0.001	
Multicenter	1	194	–	1.45(1.07-1.97)	0.018	–	–	
Cut-off value								0.615
<1.1	4	665	Fixed	1.31(1.16-1.47)	<0.001	0	0.760	
≥1.1	4	1,343	Random	1.94(1.36-2.76)	<0.001	85.5	<0.001	
Cut-off selection								0.339
ROC curve	7	1,605	Fixed	1.43(1.31-1.56)	<0.001	1.2	0.415	
X-tile	1	403	–	3.63(2.58-5.10)	<0.001	–	–	
Survival analysis								0.706
Univariate	3	949	Random	1.98(1.13-3.49)	0.017	89.5	<0.001	
Multivariate	5	1,059	Fixed	1.42(1.29-1.56)	<0.001	32.5	0.205	

SIRI, systemic inflammation response index; PFS, progression-free survival; HCC, hepatocellular carcinoma; TACE, transcatheter arterial chemoembolization; RFA, radiofrequency ablation; LT, liver transplantation; ICIs, immune checkpoint inhibitors; ROC, receiver operating characteristic.

### The relationship of SIRI with clinicopathological characteristics of HCC

**Figure 4:**
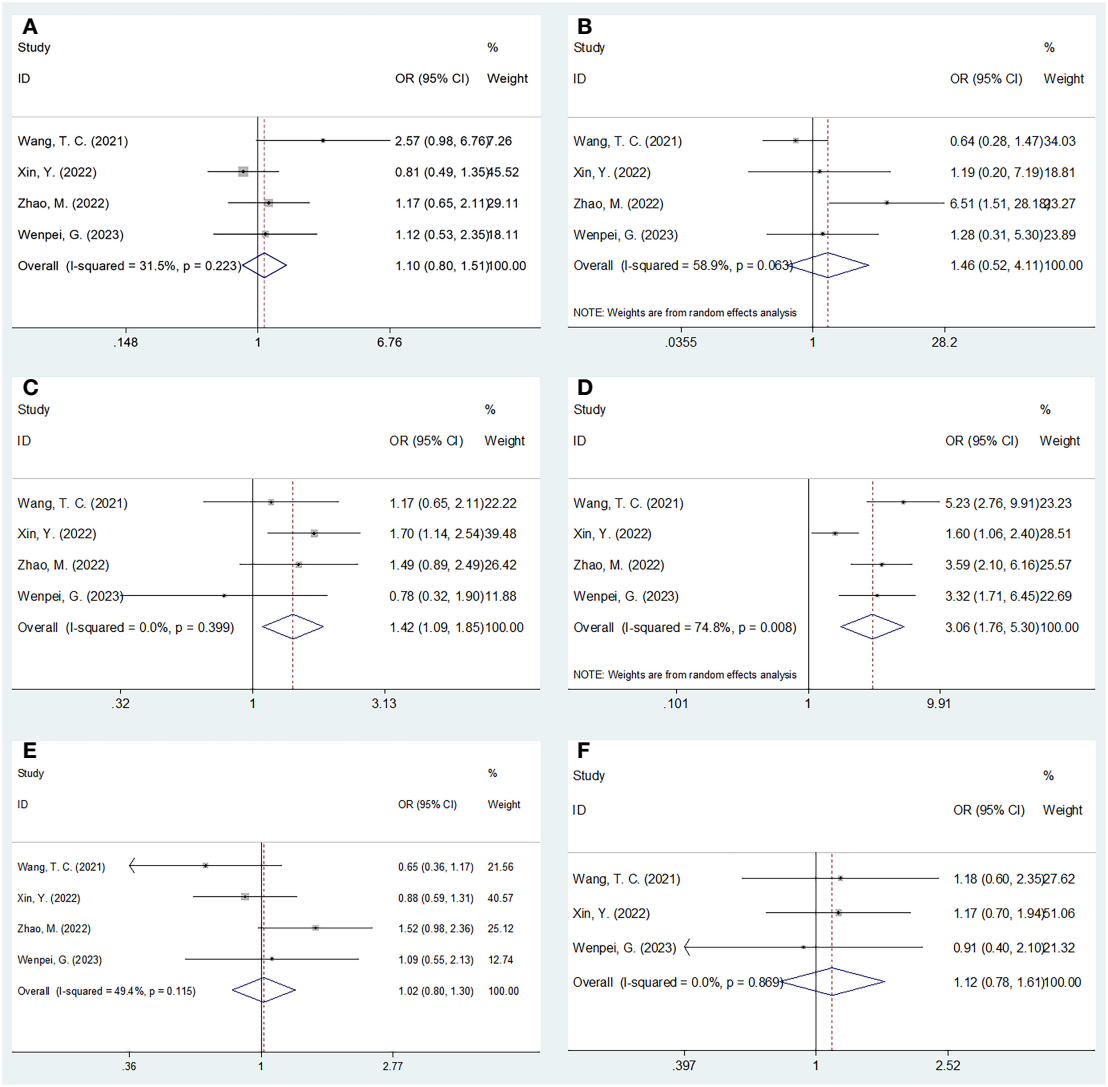
Forest plots of the associations between SIRI and clinicopathological factors in HCC. **(A)** Gender (male vs female); **(B)** Child-Pugh class (B-C vs A); **(C)** Tumor number (multiple vs solitary); **(D)** Maximum tumor diameter (>5 cm vs ≤5 cm); **(E)** AFP (ng/ml) (≥400 vs <400); and **(F)** HBV (+) (yes vs no).

Four studies involving 1,111 patients investigated the relationship between the SIRI and the clinicopathological characteristics of HCC (20, 23, 24, 27). According to **Figure 4** and **Table 4**, our combined results suggested a significant relationship between increased SIRI and multiple tumor numbers (OR=1.42, 95% CI=1.09–1.85, p=0.009) and maximum tumor diameter >5 cm (OR=3.06, 95% CI=1.76–5.30, p<0.001). Nonetheless, SIRI was not significantly related to sex (OR=1.10, 95% CI=0.88–1.51, p=0.559), Child-Pugh class (OR=1.46, 95% CI=0.52–4.11, p=0.476), alpha-fetoprotein (AFP) level (OR=1.02, 95% CI=0.80–1.30, p=0.880), or hepatitis B virus infection (OR=1.12, 95% CI=0.78–1.61, p=0.550) (**Figure 4**; **Table 4**).

**Table 4:** The association between SIRI and clinicopathological factors in patients with HCC. .

Variables	No. of studies	No. of patients	Effects model	OR (95%CI)	p	Heterogeneity I^2^(%) Ph	
Gender (male vs female)	4	1,111	Fixed	1.10(0.88-1.51)	0.559	31.5	0.223
Child-Pugh class (B-C vs A)	4	1,111	Random	1.46(0.52-4.11)	0.476	58.9	0.063
Tumor number (multiple vs solitary)	4	1,111	Fixed	1.42(1.09-1.85)	0.009	0	0.399
Maximum tumor diameter (>5 cm vs ≤5 cm)	4	1,111	Random	3.06(1.76-5.30)	<0.001	74.8	0.008
AFP (ng/ml) (≥400 vs <400)	4	1,111	Fixed	1.02(0.80-1.30)	0.880	49.4	0.115
HBV (+) (yes vs no)	3	759	Fixed	1.12(0.78-1.61)	0.550	0	0.869

SIRI, systemic inflammation response index; HCC, hepatocellular carcinoma; AFP, alpha-fetoprotein; HBV, hepatitis B virus.

### Sensitivity analysis and meta-regression

**Figure 5:**
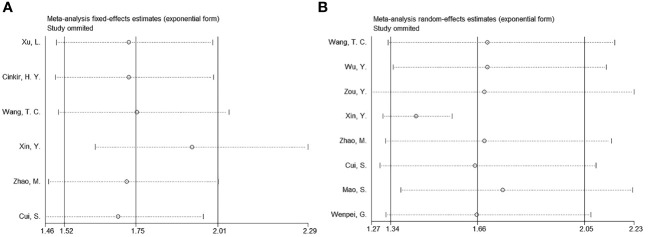
Sensitivity analysis. **(A)** OS and **(B)** PFS.

By removing studies study-by-study, sensitivity analysis revealed that none of the studies had an effect on OS or PFS, suggesting that all results remained consistent (**Figure 5**). Meta-regression showed that none of the factors significantly influenced the overall results of OS and PFS (**Tables 2**, **3**).

### Publication bias

**Figure 6:**
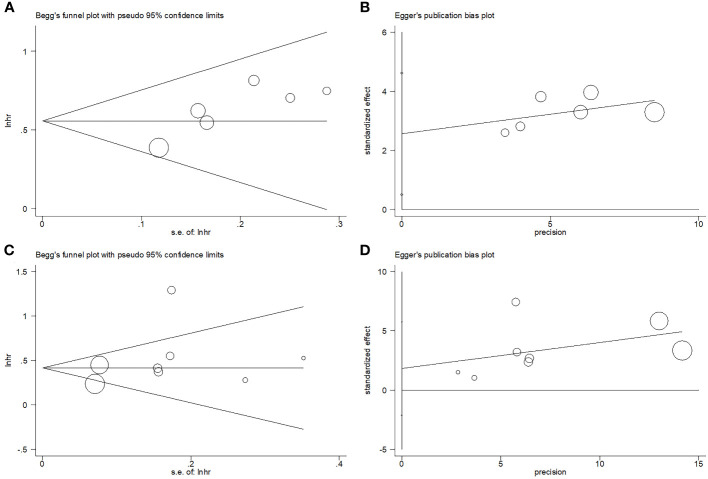
Publication test by Begg’s test and Egger’s test. **(A)** Begg’s test for OS, p=0.133; **(B)** Egger’s test for OS, p=0.358; **(C)** Begg’s test for PFS, p=0.536; and **(D)** Egger’s test for PFS, p=0.302.

Begg’s test, funnel plots, and Egger’s test were used to assess potential publication bias. The funnel plot did not exhibit any significant asymmetry in the OS or PFS (**Figure 6**). Moreover, the results indicated no significant publication bias for OS (Begg’s p=0.133; Egger’s p=0.358) or PFS (Begg’s p=0.536; Egger’s p=0.302).

## Discussion

The prognostic significance of SIRI in patients with HCC remains inconsistent. This meta-analysis collected data from 10 articles involving 2,439 patients. According to our results, elevated SIRI levels were markedly associated with shortened OS and inferior PFS in patients with HCC. Additionally, a high SIRI was significantly correlated with multiple tumor numbers and tumor size >5 cm in HCC. Publication bias tests and subgroup analyses were conducted to verify the reliability of the findings. Collectively, SIRI serves as a promising and cost-effective factor for predicting the short- and long-term prognoses of patients with HCC. To the best of our knowledge, this is the first meta-analysis to explore the potential of SIRI in predicting the prognosis of patients with HCC.

SIRI was calculated using the following formula: SIRI= neutrophil count × monocyte count/lymphocyte count. Therefore, a high SIRI could be the result of increased neutrophils, increased monocytes, and/or decreased lymphocytes. Currently, the accurate predictive mechanism of SIRI in HCC prognosis remains largely unclear and can be interpreted as follows: First, neutrophils recruited to a tumor site can produce inflammatory factors, such as interleukin-1 (IL-1) and IL-6, promoting the growth and metastasis of tumor cells (7). Activated neutrophils can suppress T cell proliferation and cytotoxicity by binding PD-L1 on the neutrophil surface to PD-1 on the T cell surface (30). This may promote tumor immune evasion and malignant growth, ultimately resulting in a shorter lifespan in cancer patients (31). Second, monocytes may differentiate into tumor-associated macrophages (TAMs) (32). TAMs secrete several inflammatory factors that affect the tumor microenvironment, thereby promoting tumor occurrence, metastasis, and relapse (33). Many studies have demonstrated the presence of TAM infiltration in the HCC matrix, accelerating tumor angiogenesis, growth, metastasis, and immunosuppression (34, 35). Third, lymphocytes play a critical role in cellular anti-tumor responses. Tumor-infiltrating lymphocytes (TILs) have a pivotal impact on the anticancer immune microenvironment and are involved in multiple stages of tumor progression (36). The presence of lymphocyte infiltration in tumor tissues is associated with improved therapeutic outcomes. However, when the number of lymphocytes in the tumor microenvironment decreases, anti-tumor ability decreases, resulting in immune tolerance and tumor escape (37). Taken together, a high SIRI may significantly predict the prognosis of patients with HCC.

Notably, the degree of liver fibrosis may significantly affect SIRI. As these data were rarely evaluated in the included papers, they were not analyzed. However, the development of HCC in cirrhotic and non-cirrhotic livers differs significantly. Future studies are needed to investigate the prognostic value of SIRI for HCC in both cirrhotic and non-cirrhotic liver groups. Evaluating liver function is essential for determining the prognosis of HCC although the Child-Pugh score alone is not sufficient to do so. A major prognostic factor for HCC is portal hypertension, which may affect the SIRI; however, this has rarely been examined in the included studies. None of the included studies provided data on portal hypertension. Therefore, we expect future studies to evaluate the prognostic value of the SIRI under different circumstances of portal hypertension. Sarcopenia is another important prognostic factor in patients with solid tumors (38–42). Current evidence shows that sarcopenia is independently associated with a poor prognosis in various cancers (38–42). Previous studies have also indicated that many patients with HCC have sarcopenia (43–45). The SIRI is associated with tumor characteristics but may also be influenced by general health status, particularly sarcopenia. However, the included studies did not present data on sarcopenia. Therefore, we expect that the correlation between SIRI and sarcopenia in HCC can be investigated in future studies.

Recently, meta-analyses have been conducted to determine whether SIRI can be used to predict the prognosis of solid tumors. A meta-analysis conducted by Wang et al. found that the SIRI independently predicted the prognosis and survival status of nasopharyngeal carcinoma based on 3,187 patients (46). Another meta-analysis of 10,754 cases by Zhou et al. showed that a high SIRI was related to shorter OS and DFS/recurrence-free survival/PFS in various cancers (47). In addition, in a meta-analysis of 14 studies, Wei et al. demonstrated that the SIRI is a useful factor for predicting dismal prognostic outcomes during malignancy treatment (48). The present study identified an obvious relationship between SIRI and survival in HCC, which is in accordance with findings in other cancer types.

This study has certain limitations. First, the articles included were from Asian countries, particularly China. Although only articles in the English language were included, the applicability of our results should be noted. Second, the SIRI cut-off values differed, possibly inducing heterogeneity in the present study. Third, the sample size was relatively small. Therefore, large-scale prospective trials using standard SIRI cut-off values should be conducted for further validation.

## Conclusions

In conclusion, the present study showed that elevated SIRI levels significantly predicted OS and PFS in patients with HCC. Moreover, the SIRI was significantly associated with tumor aggressiveness. The SIRI could be used as a promising prognostic index for HCC in clinical practice.

## Data availability statement

The original contributions presented in the study are included in the article/**Supplementary Material**. Further inquiries can be directed to the corresponding author.

## Author contributions

SZ: Conceptualization, Formal analysis, Investigation, Methodology, Project administration, Resources, Supervision, Validation, Writing – original draft. ZT: Conceptualization, Data curation, Formal analysis, Investigation, Methodology, Project administration, Resources, Supervision, Validation, Visualization, Writing – review & editing.

## Supplementary material

SUPPLEMENTARY DATA SHEET 1 The detailed search strategies of each database in this meta-analysis.
