# Supplementary material for: Prognostic and clinicopathological significance of systemic inflammation response index in patients with hepatocellular carcinoma: a systematic review and meta-analysis
Source: Front Immunol. 2024 Feb 26;15:1291840. doi: 10.3389/fimmu.2024.1291840 (PMC10925676; doi:10.3389/fimmu.2024.1291840)
Supplement: SUPPLEMENTARY DATA SHEET 1 — The detailed search strategies of each database in this meta-analysis. [file DataSheet_1.docx]

**Supplemental file 1 The detailed search strategies of each database in this meta-analysis.**

1. **Search strategies for PubMed**

Search: (systemic inflammation response index or system inflammation response index or SIRI or systemic inflammatory response index) and (hepatocellular carcinoma or hepatocellular cancer or HCC or liver cancer) Sort by: Most Recent

((("systemic"[All Fields] OR "systemically"[All Fields] OR "systemics"[All Fields]) AND ("inflammation"[MeSH Terms] OR "inflammation"[All Fields] OR "inflammations"[All Fields] OR "inflammation s"[All Fields]) AND ("response"[All Fields] OR "responses"[All Fields] OR "responsive"[All Fields] OR "responsiveness"[All Fields] OR "responsivenesses"[All Fields] OR "responsives"[All Fields] OR "responsivities"[All Fields] OR "responsivity"[All Fields]) AND ("abstracting and indexing"[MeSH Terms] OR ("abstracting"[All Fields] AND "indexing"[All Fields]) OR "abstracting and indexing"[All Fields] OR "index"[All Fields] OR "indexed"[All Fields] OR "indexes"[All Fields] OR "indexing"[All Fields] OR "indexation"[All Fields] OR "indexations"[All Fields] OR "indexe"[All Fields] OR "indexer"[All Fields] OR "indexers"[All Fields] OR "indexs"[All Fields])) OR (("system"[All Fields] OR "system s"[All Fields] OR "systems"[All Fields]) AND ("inflammation"[MeSH Terms] OR "inflammation"[All Fields] OR "inflammations"[All Fields] OR "inflammation s"[All Fields]) AND ("response"[All Fields] OR "responses"[All Fields] OR "responsive"[All Fields] OR "responsiveness"[All Fields] OR "responsivenesses"[All Fields] OR "responsives"[All Fields] OR "responsivities"[All Fields] OR "responsivity"[All Fields]) AND ("abstracting and indexing"[MeSH Terms] OR ("abstracting"[All Fields] AND "indexing"[All Fields]) OR "abstracting and indexing"[All Fields] OR "index"[All Fields] OR "indexed"[All Fields] OR "indexes"[All Fields] OR "indexing"[All Fields] OR "indexation"[All Fields] OR "indexations"[All Fields] OR "indexe"[All Fields] OR "indexer"[All Fields] OR "indexers"[All Fields] OR "indexs"[All Fields])) OR "SIRI"[All Fields] OR (("systemic"[All Fields] OR "systemically"[All Fields] OR "systemics"[All Fields]) AND ("inflammatories"[All Fields] OR "inflammatory"[All Fields]) AND ("response"[All Fields] OR "responses"[All Fields] OR "responsive"[All Fields] OR "responsiveness"[All Fields] OR "responsivenesses"[All Fields] OR "responsives"[All Fields] OR "responsivities"[All Fields] OR "responsivity"[All Fields]) AND ("abstracting and indexing"[MeSH Terms] OR ("abstracting"[All Fields] AND "indexing"[All Fields]) OR "abstracting and indexing"[All Fields] OR "index"[All Fields] OR "indexed"[All Fields] OR "indexes"[All Fields] OR "indexing"[All Fields] OR "indexation"[All Fields] OR "indexations"[All Fields] OR "indexe"[All Fields] OR "indexer"[All Fields] OR "indexers"[All Fields] OR "indexs"[All Fields]))) AND ("carcinoma, hepatocellular"[MeSH Terms] OR ("carcinoma"[All Fields] AND "hepatocellular"[All Fields]) OR "hepatocellular carcinoma"[All Fields] OR ("hepatocellular"[All Fields] AND "carcinoma"[All Fields]) OR ("liver neoplasms"[MeSH Terms] OR ("liver"[All Fields] AND "neoplasms"[All Fields]) OR "liver neoplasms"[All Fields] OR ("hepatocellular"[All Fields] AND "cancer"[All Fields]) OR "hepatocellular cancer"[All Fields]) OR "HCC"[All Fields] OR ("liver neoplasms"[MeSH Terms] OR ("liver"[All Fields] AND "neoplasms"[All Fields]) OR "liver neoplasms"[All Fields] OR ("liver"[All Fields] AND "cancer"[All Fields]) OR "liver cancer"[All Fields]))

Translations

systemic: "systemic"[All Fields] OR "systemically"[All Fields] OR "systemics"[All Fields]

inflammation: "inflammation"[MeSH Terms] OR "inflammation"[All Fields] OR "inflammations"[All Fields] OR "inflammation's"[All Fields]

response: "response"[All Fields] OR "responses"[All Fields] OR "responsive"[All Fields] OR "responsiveness"[All Fields] OR "responsivenesses"[All Fields] OR "responsives"[All Fields] OR "responsivities"[All Fields] OR "responsivity"[All Fields]

index: "abstracting and indexing"[MeSH Terms] OR ("abstracting"[All Fields] AND "indexing"[All Fields]) OR "abstracting and indexing"[All Fields] OR "index"[All Fields] OR "indexed"[All Fields] OR "indexes"[All Fields] OR "indexing"[All Fields] OR "indexation"[All Fields] OR "indexations"[All Fields] OR "indexe"[All Fields] OR "indexer"[All Fields] OR "indexers"[All Fields] OR "indexs"[All Fields]

system: "system"[All Fields] OR "system's"[All Fields] OR "systems"[All Fields]

inflammation: "inflammation"[MeSH Terms] OR "inflammation"[All Fields] OR "inflammations"[All Fields] OR "inflammation's"[All Fields]

response: "response"[All Fields] OR "responses"[All Fields] OR "responsive"[All Fields] OR "responsiveness"[All Fields] OR "responsivenesses"[All Fields] OR "responsives"[All Fields] OR "responsivities"[All Fields] OR "responsivity"[All Fields]

index: "abstracting and indexing"[MeSH Terms] OR ("abstracting"[All Fields] AND "indexing"[All Fields]) OR "abstracting and indexing"[All Fields] OR "index"[All Fields] OR "indexed"[All Fields] OR "indexes"[All Fields] OR "indexing"[All Fields] OR "indexation"[All Fields] OR "indexations"[All Fields] OR "indexe"[All Fields] OR "indexer"[All Fields] OR "indexers"[All Fields] OR "indexs"[All Fields]

systemic: "systemic"[All Fields] OR "systemically"[All Fields] OR "systemics"[All Fields]

inflammatory: "inflammatories"[All Fields] OR "inflammatory"[All Fields]

response: "response"[All Fields] OR "responses"[All Fields] OR "responsive"[All Fields] OR "responsiveness"[All Fields] OR "responsivenesses"[All Fields] OR "responsives"[All Fields] OR "responsivities"[All Fields] OR "responsivity"[All Fields]

index: "abstracting and indexing"[MeSH Terms] OR ("abstracting"[All Fields] AND "indexing"[All Fields]) OR "abstracting and indexing"[All Fields] OR "index"[All Fields] OR "indexed"[All Fields] OR "indexes"[All Fields] OR "indexing"[All Fields] OR "indexation"[All Fields] OR "indexations"[All Fields] OR "indexe"[All Fields] OR "indexer"[All Fields] OR "indexers"[All Fields] OR "indexs"[All Fields]

hepatocellular carcinoma: "carcinoma, hepatocellular"[MeSH Terms] OR ("carcinoma"[All Fields] AND "hepatocellular"[All Fields]) OR "hepatocellular carcinoma"[All Fields] OR ("hepatocellular"[All Fields] AND "carcinoma"[All Fields])

hepatocellular cancer: "liver neoplasms"[MeSH Terms] OR ("liver"[All Fields] AND "neoplasms"[All Fields]) OR "liver neoplasms"[All Fields] OR ("hepatocellular"[All Fields] AND "cancer"[All Fields]) OR "hepatocellular cancer"[All Fields]

liver cancer: "liver neoplasms"[MeSH Terms] OR ("liver"[All Fields] AND "neoplasms"[All Fields]) OR "liver neoplasms"[All Fields] OR ("liver"[All Fields] AND "cancer"[All Fields]) OR "liver cancer"[All Fields]

1. **Search strategies for Web of Science:**

(systemic inflammation response index or system inflammation response index or SIRI or systemic inflammatory response index) and (hepatocellular carcinoma or hepatocellular cancer or HCC or liver cancer) (all fields)

1. **Search strategies for Embase:**

#2 #1 AND 'article'/it

#1 ('systemic inflammation response index'/exp OR 'systemic inflammation response index' OR (systemic AND ('inflammation'/exp OR inflammation) AND ('response'/exp OR response) AND ('index'/exp OR index)) OR 'system inflammation response index' OR (system AND ('inflammation'/exp OR inflammation) AND ('response'/exp OR response) AND ('index'/exp OR index)) OR 'siri'/exp OR siri OR 'systemic inflammatory response index'/exp OR 'systemic inflammatory response index' OR (systemic AND inflammatory AND ('response'/exp OR response) AND ('index'/exp OR index))) AND ('hepatocellular carcinoma'/exp OR 'hepatocellular carcinoma' OR (hepatocellular AND ('carcinoma'/exp OR carcinoma)) OR 'hepatocellular cancer'/exp OR 'hepatocellular cancer' OR (hepatocellular AND ('cancer'/exp OR cancer)) OR hcc OR 'liver cancer'/exp OR 'liver cancer' OR (('liver'/exp OR liver) AND ('cancer'/exp OR cancer)))

1. **Search strategies for Cochrane Library:**

(systemic inflammation response index or system inflammation response index or SIRI or systemic inflammatory response index) and (hepatocellular carcinoma or hepatocellular cancer or HCC or liver cancer) in Title Abstract Keyword
